# Glucose Tolerance and lipid profile changes after surgical menopause

**Published:** 2014

**Authors:** Shahla Yazdani, Majid Sharbatdaran, Mohammad Abedi Samakoosh, Zinatossadat Bouzari, Zahra Masoudi

**Affiliations:** 1 Fatemeh Zahra Infertility and Reproductive Health Research Center, Babol University of Medical Sciences , Babol, Iran.; 2 Department of Gynacology, Babol University of Medical Sciences, Babol, Iran.; 3 Department of Pathology , babol University of Medical Sciences, babol , Iran.; 4 Department of Internal Medicine, Mazandaran University of Medical Sciences, Sari, Iran.; 5 Cellular and Molecular Biology Research Center, Babol University of Medical Sciences. Babol, Iran.; 6 Babol University of Medical Sciences, Babol, Iran.

**Keywords:** Salpingo-oophorectomy, Menopause, Glucose tolerance, Lipid profile

## Abstract

***Background: ***Bilateral salpingo-oophorectomy in women may lead to metabolic consequences in patients. This study was performed to determine the glucose tolerance and lipid profile after menopause resulting from bilateral salpingo-oophorectomy.

***Methods:*** From September 2011 to March 2013, 31 women participants aged 46-52 years who underwent hysterectomy and bilateral salpingo-oophorectomy for benign reasons were recruited. An oral glucose tolerance test (OGTT), triglyceride (TG), total cholesterol, low density lipoproteis (LDL), high density lipoprotein (HDL) was performed before and 6 months after surgery. Fasting insulin and 2-hour insulin levels, fasting insulin / glucose indexes and homeostasis model assessment HOMA indexes were also measured.

***Results:*** The mean age of the patients was 48.6±2.9 years. The mean 2-h glucose level in OGTT changed from 26.7 before surgery to 111.1 µUnit/ml (P=0.030). The mean level of lipid profile before and after surgery for TG was 132.3 versus 181.2 mg/dl (P=0.005), total cholesterol 177.4 versus 206.7 mg/dl (P=0.0001) and LDL 98.4 versus 115.3 mg/dl (P=0.003). The other variables showed no significant difference.

***Conclusion: ***The results indicate that lipid profile changes like increase of TG, total cholesterol and LDL should be considered before removing the ovary during hysterectomy in premenopausal women.

The prevalence of diabetes type II and impaired glucose tolerance increase after age 40 (1). It is believed that decreased estrogen level and genetic predisposition are responsible for these changes in premenopause stage. Some studies have evaluated the metabolism of insulin after menopause (2). Also, the effect of hormone replacement therapy on glucose metabolism in postmenopausal stage has been shown in some studies (3-6). In a study in Turkey in 2011, it was demonstrated that menopause after bilateral salpingo-oophorectomy would result in impaired glucose metabolism (7). In a study in 2003, low-dose hormone-therapy could increase insulin sensitivity in healthy menopause women (8). Another study revealed that tibolone (a systemic steroid) would increase peripheral sensitivity to insulin leading to improved lipid profile (9). In a study by Tuna in 2007, comparing lipid profile in menopause women due to surgery and those resulted from normal menopause it was seen that HDL and VLDL (very low density lipoprotein) levels are lower and higher in those with surgical menopause, respectively (10). In some current studies, also the lipid profile in different times after hysterectomy and bilateral salpingo-oophorectomy (from eight weeks to six months) were evaluated, the changes in each lipid level in various time intervals showed different results (11-13).

But in studies in the stages of menopause, the development of side effects such as osteoporosis, urogenital atrophy, etc in early menopausal ages and increased late cardiovascular and neurological complications due to vascular and metabolic alterations are demonstrated (14). This study was performed to determine the glucose tolerance and lipid profile after menopause resulting from bilateral salpingo-oophorectomy.

## Methods

This study was performed among 35 women aged from 46-52 years that were candidate for hysterectomy and bilateral salpingo-oophorectomy due to benign diseases such as pelvic endometriosis,chronic pelvic pain, ovarian cancer prophylaxis, and severe dysmenorrheal during September 2011 to March 2013. In two patients, the surgery was cancelled (one because of the patient's decision and another due to pulmonary problems); one had follicular stimulating hormone (FSH) higher than 30 (mIU/ml) and one lost to follow-up. The inclusion criteria were age range 46 to 52 years, hysterectomy and bilateral salpingo-oophorectomy for benign causes, and lack of background disease history. The exclusion criteria include a body mass index (BMI) more than 40 kg/m² and the FSH level higher than 30 (mIU/ml). FSH, two-hour tolerance test, fasting and two-hour insulin, and lipid profile (cholesterol, TG, LDL, HDL) were measured in a single lab in baseline and after six months from operation. The tests in baseline and final measurements were compared. OGTT with 75 gram glucose after fasting during night was performed and was considered normal if 2-hpG < 7.8 mmol/L (140 mg/dL) and fasting plasma glucose (FPG) < 5.6 mmol/L (100 mg/dL). The impaired glucose test was defined as FPG: 5.6-6.9 mmol/L (100-125 mg/dL) or 2-hpG = 7.8 – 11 mmol/L (140-199 mg/dl). The higher levels were considered as diabetes type two. In addition, both FPG and 2h pG the insulin were also measured. Likewise, the insulin/glucose index and HOMA (homeostasis model assessment) were calculated at baseline and after six months, if insulin/glucose index was higher than 22 and HOMA was more than four, this was considered as impaired glucose tolerance.

The data were collected and analyzed with SPSS version 13.0 (Chicago, Illinois, USA). Paired-sample t-test was used for comparison between before and after phases and was considered statistically significant at p-value less than 0.05.

## Results

Thirty premenopausal patients entered in to this study with the mean age of 48.1±2.9 years and median of parity was 3±1.4, the mean of BMI was 28±4.3 kg/m. As shown in table1, the GTT showed significant reduction (P=0.030), and FSH (P=0.0001), TG (P=0.005), total cholesterol (P=0.0001), and LDL (P=0.003) showed significant increase after six months. The other factors were not significantly different (P>0.05).

**Table 1:** Before and after measurements in patients

**Index**	**Baseline**	**Six Months**	**Pvalue**
FSH (mIU/ml)	12.5	96.5	0.0001
FBS (mg/dL)	89.4	92.6	> 0.05
GTT (mg/dL)	126.7	111.1	0.030
Fasting Insulin (µUnit/ml)	11.1	11.9	> 0.05
2-Hour Insulin (µUnit/ml)	59.4	52.3	> 0.05
TG (mg/dl)	132.3	181.2	0.005
Total Cholesterol (mg/dl)	177.4	206.7	0.0001
HDL Cholesterol (mg/dl)	51	53.9	> 0.05
LDL Cholesterol (mg/dl)	98.4	115.3	0.003
HOMA	2.5	2.8	> 0.05
Fasting Insulin/Glucose	2.2	2.3	> 0.05

## Discussion

The results in this cohort study comparing the glucose tolerance and lipid profile at baseline and after six months from hysterectomy and bilateral salpingo-oophorectomy showed that the TG, total cholesterol, and LDL had significant increase and the HDL slightly increased without significant difference. The GTT had significant decrease but HOMA and insulin/glucose ratio showed increase to decrease insulin resistance but without significant difference. Yoshida et al. evaluated the premenopausal women in two groups. Twenty-seven subjects with ovarian preservation and 35 with bilateral oophorectomy. They found a significant elevation in the level of LDL in the latter group, but the level did not change in the former group. Regarding carbohydrate metabolism, the result was similar to the finding of our study (15). In two different studies with bilateral salpingo-oophorectomy patients in Italy showed increased total cholesterol and LDL levels that were similar to the findings of our study (16, 6). Cheung et al. (12) evaluated 100 patients without any significant difference after six months; however, the changes were significant after eight weeks. But we found significant results after six months. Casiglia et al in Italy showed significant increase only about TG contrary to our results that showed significant increase in total and LDL cholesterols (13). The study by Kabir et al. compared 30 women with normal and surgical menopause and showed that TG was higher and the LDL cholesterol was lower in surgical group (17). Similar study was performed by Tuna et al in Turkey among two the groups of 50 subjects each with lower HDL and higher VLDL cholesterols in surgical group (10). 

Pirimoglu et al in Turkey found that mean fasting glucose and two-hour results had no significant change but the glucose tolerance was impaired and the insulin response to glucose tolerance test had significant increase and the insulin index was changed slowly during 12 months after surgical menopause. They concluded that increased insulin secretion might be the cause of lower glucose level (7). In our study, also fasting glucose had no significant change but was significantly decreased after two hours. The two-hour insulin level showed non-significant decrease in our study and the insulin to glucose index and HOMA-IR had no significant change and we found two cases of impaired GTT and FBS and no case of diabetes. The reason for this difference may be a longterm (12 months) follow up in their study. Totally, according to our results, the lipid profile of women under bilateral salpingo-oophorectomy and hysterectomy showed significant increase in triglyceride, total cholesterol and LDL cholesterol after six months. The low number of our cases, lack of measurement of the body weight (BMI) and small duration of follow up may be the weakness of this study.

We found no evidence of glucose intolerance. Further studies with longterm follow-up and large population based sample are recommended to evaluate the probability of glucose intolerance results.

## Notes

**Funding:** We would like to thank the Vice Chancellor, for Research and Technology of Babol University of Medical Sciences for the approval of this study (Grant Number: 9032533).
